# Efficient Plasma and Placenta Metabolic Fingerprints for Preeclampsia Diagnosis: A Multicenter Study

**DOI:** 10.1002/mco2.71139

**Published:** 2026-11-08

**Authors:** Yetao Xu, Yuchen Liu, Kun Xu, Xiaokang Deng, Kejia Cao, Junjie Guo, Jiao Wu, Juxiang Zhang, Yanxi Yang, Yanyan Li, Huihui Ju, Xiaoyan Zhou, Weichun Tang, Ying Jiang, Yida Huang, Yifan Lian, Kun Qian, Zhiwei Liu

**Affiliations:** ^1^ Department of Obstetrics and Gynecology School of Medicine International Peace Maternity and Child Health Hospital, Shanghai Jiao Tong University Shanghai China; ^2^ Shanghai Key Laboratory of Embryo Original Diseases Shanghai China; ^3^ Department of Obstetrics and Gynecology First Affiliated Hospital With Nanjing Medical University Nanjing China; ^4^ State Key Laboratory of Systems Medicine for Cancer School of Biomedical Engineering, Institute of Medical Robotics and Shanghai Academy of Experimental Medicine, Shanghai Jiao Tong University Shanghai China; ^5^ Division of Cardiology Renji Hospital, School of Medicine, Shanghai Jiao Tong University Shanghai China; ^6^ Department of Oncology Jiangsu Cancer Hospital, The Affiliated Cancer Hospital of Nanjing Medical University, Jiangsu Institute of Cancer Research Nanjing China; ^7^ Wuxi Maternity and Child Health Care Hospital, Affiliated Women's Hospital of Jiangnan University Wuxi China; ^8^ Changzhou Maternal and Child Health Care Hospital, Nanjing Medical University Changzhou China; ^9^ Department of Obstetrics The Affiliated Huai'an No. 1 People's Hospital of Nanjing Medical University Huaian China; ^10^ Department of Obstetrics Affiliated Hospital of Nantong University, Nantong First People's Hospital Nantong China; ^11^ Department of Obstetrics Women's Hospital, Zhejiang University School of Medicine Hangzhou China; ^12^ Department of Gastroenterology The Second Affiliated Hospital of Fujian Medical University Quanzhou China

**Keywords:** biomarker, diagnosis, preeclampsia, placenta metabolic fingerprints, plasma metabolic fingerprints

## Abstract

Preeclampsia (PE) is a leading cause of maternal and perinatal mortality. There remains a clinical need for accurate and accessible diagnostic approaches to facilitate timely identification of PE. Herein, the nanoparticle‐enhanced laser desorption/ionization mass spectrometry is applied to obtain plasma (*n* = 236) and placenta (*n* = 70) metabolic fingerprints from a multicenter dataset (109 PE patients and 127 non‐PE participants, 4 centers). Diagnostic models are constructed with an efficient diagnostic area under the curve (AUC) of 0.941 with external validation. Subsequently, 19 PE‐associated metabolites are annotated, demonstrating good diagnostic performance with an AUC of 0.871. Moreover, diagnostic scores of severe PE patients are significantly higher than those of PE patients, exhibiting our model's capacity to differentiate disease severity. Related metabolic pathways are revealed to inform potential therapeutic strategies for PE and biological validation on trophoblast cells demonstrates that key biomarkers, including L‐alanine, taurine, hypoxanthine, and orotic acid, can significantly enhance migration capabilities (*p* < 0.01). This work highlights the potential of metabolic analysis for PE detection, paving the way for improved clinical decision‐making in obstetrics.

## Graphical abstract

Plasma and placental tissue metabolic fingerprints were obtained from a multicenter cohort using nanoparticle‐enhanced laser desorption/ionization mass spectrometry. Diagnostic models are constructed with an efficient diagnostic AUC of 0.941 with external validation. A total of 19 PE‐associated metabolites, including L‐alanine, taurine, hypoxanthine, and orotic acid, are annotated, demonstrating good diagnostic performance with an AUC of 0.871. Related metabolic pathways are revealed to inform potential therapeutic strategies of PE.

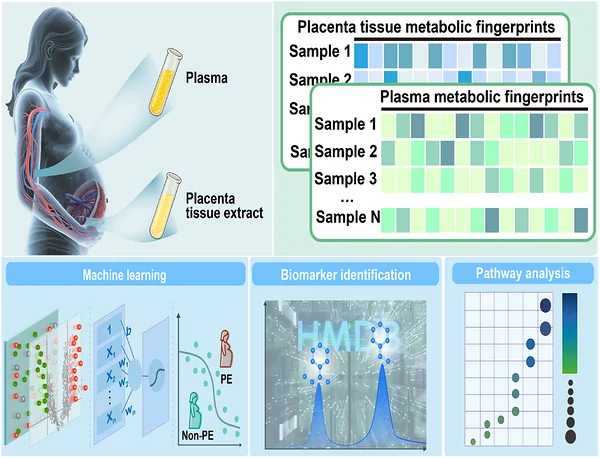

## 1 Introduction

Preeclampsia (PE), a life‐threatening pregnancy‐specific syndrome, affects 2%–8% of pregnancies globally and is a leading cause of maternal and perinatal mortality [1]. Clinically, PE is diagnosed after 20 weeks’ gestation based on new‐onset hypertension accompanied by proteinuria or end‐organ dysfunction [2]. Early identification of PE has been shown to reduce maternal morbidity through timely planned delivery [3]. Current clinical diagnostic criteria for PE include new‐onset hypertension and rapid assessment of proteinuria using the urine protein‐to‐creatinine ratio. However, 24‐h urine protein quantification suffers from poor patient adherence (>30% timing errors) [4, 5], posing a particular burden on pregnant individuals [2, 6], and risks protein degradation due to improper storage [7, 8]. These issues collectively contribute to unstable diagnostic performance, with highly variable sensitivity (50%–75%) and specificity (50%–90%) [1, 9, 10, 11]. More importantly, biomarkers capable of identifying PE risk before the onset of overt clinical manifestations may offer greater clinical value by enabling earlier risk stratification and intervention. These limitations highlight an unmet clinical need for rapid, reliable, and minimally invasive diagnostic tools that can support early triage and risk stratification in suspected PE.

Biomarker‐based blood tests have been increasingly incorporated into clinical practice due to their noninvasiveness, cost‐effectiveness, and desirable patient compliance [12, 13, 14]. Several circulating biomarkers, including soluble fms‐like tyrosine kinase‐1 (sFlt‐1), placental growth factor (PlGF), and cell‐free DNA (cfDNA), have demonstrated improved diagnostic or prognostic performance compared with traditional clinical indicators [15, 16, 17]. Notably, most of them are involved in upstream molecular events at the genomic, transcriptomic, or proteomic levels. In contrast, metabolic biomarkers represent the downstream biological products that integrate genetic, environmental, and physiological influences, thereby offering a more direct and sensitive readout of disease‐related pathophysiological alterations [18, 19]. Accordingly, metabolomics has emerged as a promising approach for early detection and characterization of PE.

Despite growing interest, existing metabolomic studies in PE remain limited. Most prior investigations have relied on conventional nuclear magnetic resonance (NMR) spectroscopy or liquid/gas chromatography–mass spectrometry (LC–MS/GC–MS), and are typically constrained by small sample sizes, single‐center designs, and analysis of a single biological sample type (predominantly maternal plasma or serum) [20, 21, 22]. These approaches typically require extensive sample preparation and lengthy analytical workflows [23, 24], which may compromise throughput and scalability in clinical applications. Furthermore, the lack of multicenter validation and integrated analysis across different tissue types limits the robustness and generalizability of reported metabolic signatures [25]. In this regard, nanoparticle‐enhanced laser desorption/ionization mass spectrometry (NPELDI‐MS), leveraging engineered nanoparticles as matrix and enabling direct solid‐phase enrichment and ionization of low‐molecular‐weight metabolites from complex biofluids [26, 27, 28], represents a rapid, high‐throughput, and stable analytical approach, which is promising for multicenter study of PE [29, 30].

Herein, the present multicenter study establishes a robust NPELDI‐MS‐based metabolic fingerprinting strategy and a panel of candidate metabolic biomarkers, providing an important basis for identifying PE‐associated alterations before overt clinical manifestations. Plasma and placenta metabolic fingerprints were obtained from a multi‐center dataset (109 PE patients and 127 non‐PE participants, 4 centers), facilitating the discovery of robust metabolic features and subsequent construction of diagnostic models for PE. Typically, the diagnostic features and models were validated in an independent external validation dataset, showing an efficient diagnostic area under the curve (AUC) of 0.941 (95% confidence interval (CI): 0.891–0.978). Subsequently, 19 PE‐associated metabolites were putatively annotated, and related dysregulated metabolic pathways were revealed to inform potential therapeutic strategies. Further, biological validation demonstrated that key biomarkers significantly enhanced trophoblast migration capabilities (*p* < 0.01). We hope this approach serves as a powerful tool for accurate detection of PE, paving the way for improved diagnostic strategies and clinical decision‐making in obstetrics.

## 2 Results

### 2.1 Study Design

We retrospectively established a PE dataset comprising 236 subjects (109 PE patients and 127 non‐PE participants) from multiple centers across two provinces in China to facilitate the discovery of robust and clinically applicable metabolic biomarkers (Figure 1A). A sample size estimation based on the primary diagnostic endpoint was performed using the Hanley–McNeil method for AUC analysis. Assuming an expected AUC of 0.80 versus a minimum acceptable AUC of 0.70, a two‐sided α of 0.05, 80% power, and an approximately balanced allocation between PE and non‐PE participants, the minimum required sample size was estimated to be 196 participants, which was exceeded by the 236 participants included in this study. Specifically, all the participants were enrolled from January 2023 to December 2024. For PE patients, the diagnostic criteria required sustained maternal hypertension (diastolic blood pressure >90 mmHg and/or systolic blood pressure >140 mmHg) arising after 20 weeks of gestation, combined with either significant proteinuria or new‐onset systemic organ dysfunction. Proteinuria is confirmed by any of the following: random dipstick ≥ 1+ (used when quantitative measurement is unavailable), urine protein/creatinine ratio >0.3, or 24‐h urinary protein >0.3 g. In the absence of proteinuria, diagnosis requires at least one manifestation of end‐organ involvement, including placental‐fetal compromise, neurological abnormalities, hematological disturbances, renal impairment, hepatic dysfunction, cardiopulmonary complications, or gastrointestinal disorders. Additionally, the non‐PE control group comprised women with uncomplicated pregnancies, and their placental tissues were collected during delivery via cesarean section at approximately 38 weeks gestation due to fetal breech position or a scarred uterus. Gestational age at sample collection was 35.7 ± 2.7 weeks for PE patients, 38.6 ± 0.9 weeks for non‐PE participants in the discovery dataset (*p* < 0.001), and 21.7 ± 3.0 weeks for PE patients, 22.6 ± 2.7 weeks for non‐PE participants in the external validation dataset (*p* = 0.08).

**FIGURE 1:**
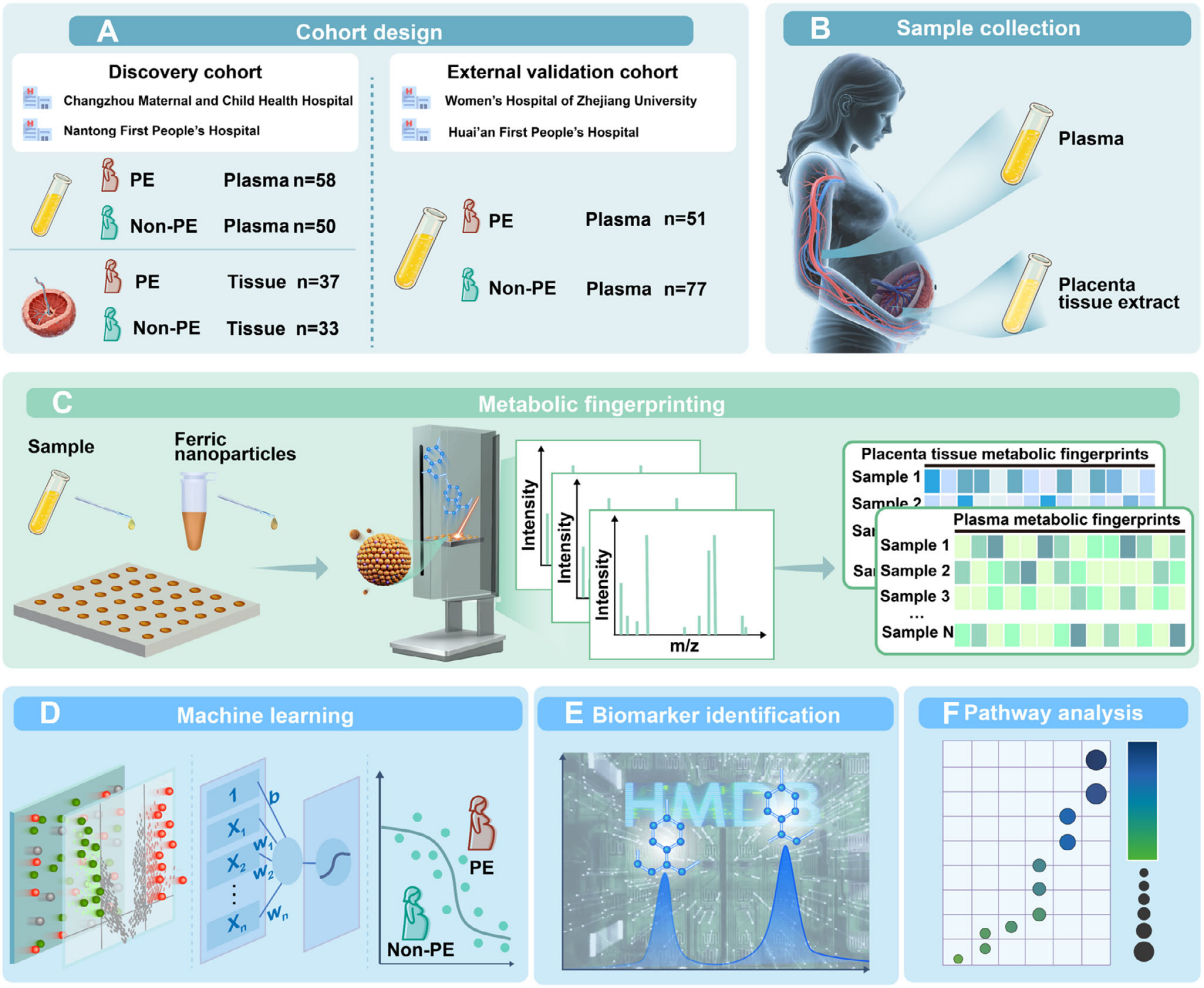
Schematic of plasma and placenta metabolic fingerprinting for preeclampsia (PE) diagnosis. (A)  Study design including a discovery dataset and an external validation dataset. (B) Plasma and placental tissue sample collection. (C) Metabolic fingerprinting workflow involves sample preparation and ferric nanoparticle deposition on microarray substrates, acquisition of raw mass spectrometric data, and generation of plasma and placenta metabolic fingerprints through data preprocessing. (D) Feature selection was conducted on metabolic fingerprinting datasets, and predictive models were developed based on machine learning approaches. (E) Biomarker annotation was conducted through Fourier transform ion cyclotron resonance mass spectrometry (FTICR‐MS) analysis, and metabolite annotation was performed based on Human Metabolome Database (HMDB) dataset. (F) PE‐associated metabolic pathways were revealed through quantitative enrichment analysis.

For biomarker discovery and model building, all the patients were stratified into a discovery dataset and an external validation dataset based on their sample sources. To be specific, the discovery dataset comprised 14 PE patients from Changzhou Maternal and Child Health Hospital and 44 PE patients plus 50 non‐PE participants from Nantong First People's Hospital, while the external validation dataset consisted of 49 non‐PE participants from the Women's Hospital of Zhejiang University and 51 PE patients plus 28 non‐PE participants from Huai'an First People's Hospital (Figure 1A). The plasma samples from all the PE patients and non‐PE participants were harvested, and particularly, 37 PE patients and 33 non‐PE participants from Nantong provided both placental tissue and plasma samples (Figure 1B).

Metabolic fingerprinting of PE‐associated plasma and placental tissue samples was conducted using NPELDI‐MS (Figure 1C), encompassing sample loading, matrix printing, MS detection, and data preprocessing. Subsequently, feature selection identified metabolic features related to PE in both placental tissue and plasma samples, and machine learning was applied to build diagnostic models based on plasma metabolic fingerprints (Figure 1D). Further, diagnostic metabolites for PE were putatively annotated (Figure 1E), and their associated pathways were elucidated to facilitate development of potential therapeutic strategies (Figure 1F).

### 2.2 Establishment of PE‐Associated Plasma and Placenta Metabolic Fingerprints

To generate a robust and reproducible dataset essential for subsequent metabolic investigations, comprehensive metabolic fingerprinting was performed using high‐performance NPELDI‐MS (Supporting Information). Our methodology typically utilized ferric nanoparticles as the matrix. These nanoparticles offered distinct advantages, including (1) nanoscale surface roughness enabling size‐selective enrichment of low‐molecular‐weight metabolites within complex biological samples (Figure S1A), (2) a monodisperse size distribution, evidenced by a polydispersity index (PDI) of approximately 0.2, ensuring uniform deposition on microarrays (Figure S1B), (3) a negatively charged surface (approximately −23.43 mV) which promoted efficient positive ionization during MS analysis (Figure S1C), and (4) strong UV laser absorption at the wavelength range of 300–450 nm, consistent with the wavelength of equipped Nd:YAG laser for NPELDI‐MS (355 nm, Figure S1D). These features enabled rapid sample analysis at 2 s/sample (using 2000 laser shots at 1000 Hz). The microarray design further achieved high‐throughput detection of 384 samples (consisting of 320 clinical samples, 40 quality control samples, and 24 calibration standards) per batch (Figure S1E). Specifically, as a quantitative characterization of NPELDI‐MS robustness, coefficients of variation (CVs) were computed for four representative metabolites, including valine (Val, [Val + Na]^+^ at *m/z* of 140.07), lysine (Lys, [Lys + Na]^+^ at *m/z* of 169.07), glucose (Glc, [Glc + Na]^+^ at *m/z* of 203.05), and sucrose (Suc, [Suc + Na]^+^ at *m/z* of 365.11), using 10 independent replicate samples. Consequently, the observed CVs spanned from 10.2% to 12.3% (Figure S1F, Table S1), demonstrating the desirable reproducibility of NPELDI‐MS. Specifically, we further benchmarked ferric nanoparticles against representative alternative NPELDI matrices, including MOF‐based materials, gold nanoparticles, and other metal oxide nanoparticles. Ferric nanoparticles showed favorable overall performance in terms of 355‐nm optical absorption, low‐mass background, ionization efficiency, reproducibility, salt tolerance, cost, and compatibility with high‐throughput detection (Figure S2).

Subsequently, we constructed PE‐associated plasma and placenta metabolic fingerprints based on NPELDI‐MS. Specifically, the mass spectra of 236 plasma samples from PE patients (n = 109) and non‐PE participants (n = 127), as well as 70 placental tissue samples from PE patients (n = 37) and non‐PE participants (n = 33), were acquired (Figure 2A), yielding ∼130,000 data points per spectrum for plasma and placental tissue within the mass range of 80–1000 Da (Figure 2B,C). After preprocessing, we extracted 351 and 430 *m/z* signals from raw spectra data of plasma and placental tissue, respectively, to construct PE‐associated metabolic fingerprints for diagnostic purposes (Figure 2D,E). The intra‐group similarity, evaluated using the cosine algorithm, showed similarity scores exceeding 0.75 in all groups, confirming the high reproducibility of the NPELDI‐MS‐based metabolic analysis (Figure 2F,G). During NPELDI‐MS analysis, quality control was performed by analyzing standard samples containing four representative metabolites—Val, Lys, Glc, and Suc—once every eight samples throughout the analytical sequence to monitor the long‐term stability of the platform. The CVs of the signal intensities for these metabolites ranged from 13.0% to 19.6%, indicating acceptable analytical stability during large‐scale metabolic profiling (Table S2). To visualize the clustering of samples based on plasma and placental tissue metabolic fingerprints, we applied three commonly used unsupervised algorithms. Although some degree of separation between PE and non‐PE groups was observed, substantial overlap remained, indicating that simple unsupervised clustering methods were insufficient for accurate PE diagnosis (Figure 2H–J). Therefore, advanced machine learning algorithms were required to achieve efficient and precise diagnostic performance.

**FIGURE 2:**
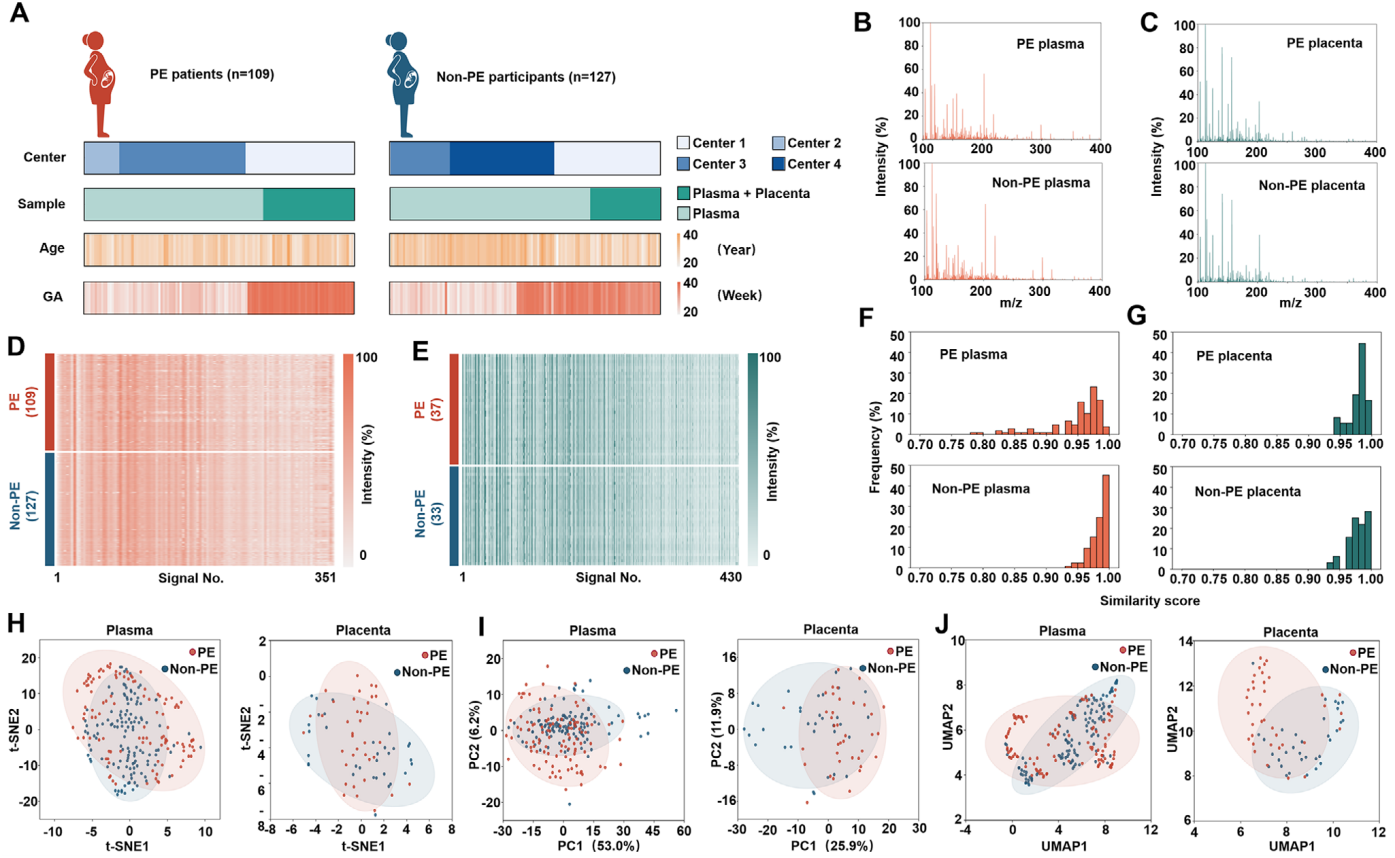
Establishment of preeclampsia (PE)‐associated plasma and placenta metabolic fingerprints. (A) The center, sample type, age and gestational age (GA) at sample collection distribution of PE patients (n = 109) and non‐PE participants (n = 127). Centers 1–4 correspond to Nantong First People's Hospital, Changzhou Maternal and Child Health Hospital, Huai'an First People's Hospital, and the Women's Hospital of Zhejiang University, respectively. (B) The mass spectra of PE and non‐PE plasma samples. (C) The mass spectra of PE and non‐PE placental samples. (D) Plasma metabolic fingerprints, including 351 *m/z* features, and (E) placenta metabolic fingerprints, including 430 *m/z* features, were extracted from the original MS data. The color scale was logarithmically adjusted. Distribution of similarity scores of (F) plasma and (G) placenta metabolic fingerprints in PE and non‐PE groups, demonstrating all the samples exhibited similarity scores exceeding 0.75. (H) T‐distributed stochastic neighbor embedding (t‐SNE) map, (I) principal component analysis (PCA) score plot, and (J) uniform manifold approximation and projection (UMAP) visualization of all samples, including PE patients (red) and non‐PE participants (blue).

### 2.3 Construction of Machine Learning Models for PE Diagnosis

To investigate the diagnostic potential of metabolic fingerprints for PE, we applied machine learning approaches to construct diagnostic models (Supporting Information). A total of 236 subjects were included and stratified into a discovery dataset and an external validation dataset based on their sample sources as described above (Tables S3 and S4).

We assessed the diagnostic performance of plasma metabolic fingerprints based on three machine learning algorithms: elastic net (EN), lasso regression (LR), and ridge regression (RR). In the discovery dataset, via receiver operating characteristic (ROC) curve analysis, these models achieved AUCs ranging from 0.932 to 0.974, demonstrating high diagnostic accuracy (Figure 3A, Table S5). Comparable performance was observed in the external validation dataset, with AUCs between 0.965 and 0.992 (Figure 3B, Table S5). Specifically, the sample‐level plot of diagnostic scores generated by three machine learning algorithms showed distinct differences between PE and non‐PE participants, further verifying effective diagnostic efficiency (Figure S3A–C). Subsequent analysis using precision–recall (PR) curve supported these findings, with areas under the PR curve (AUPRC) exceeding 0.945 across all algorithms in both discovery and external validation datasets (Figure S3D,E).

**FIGURE 3:**
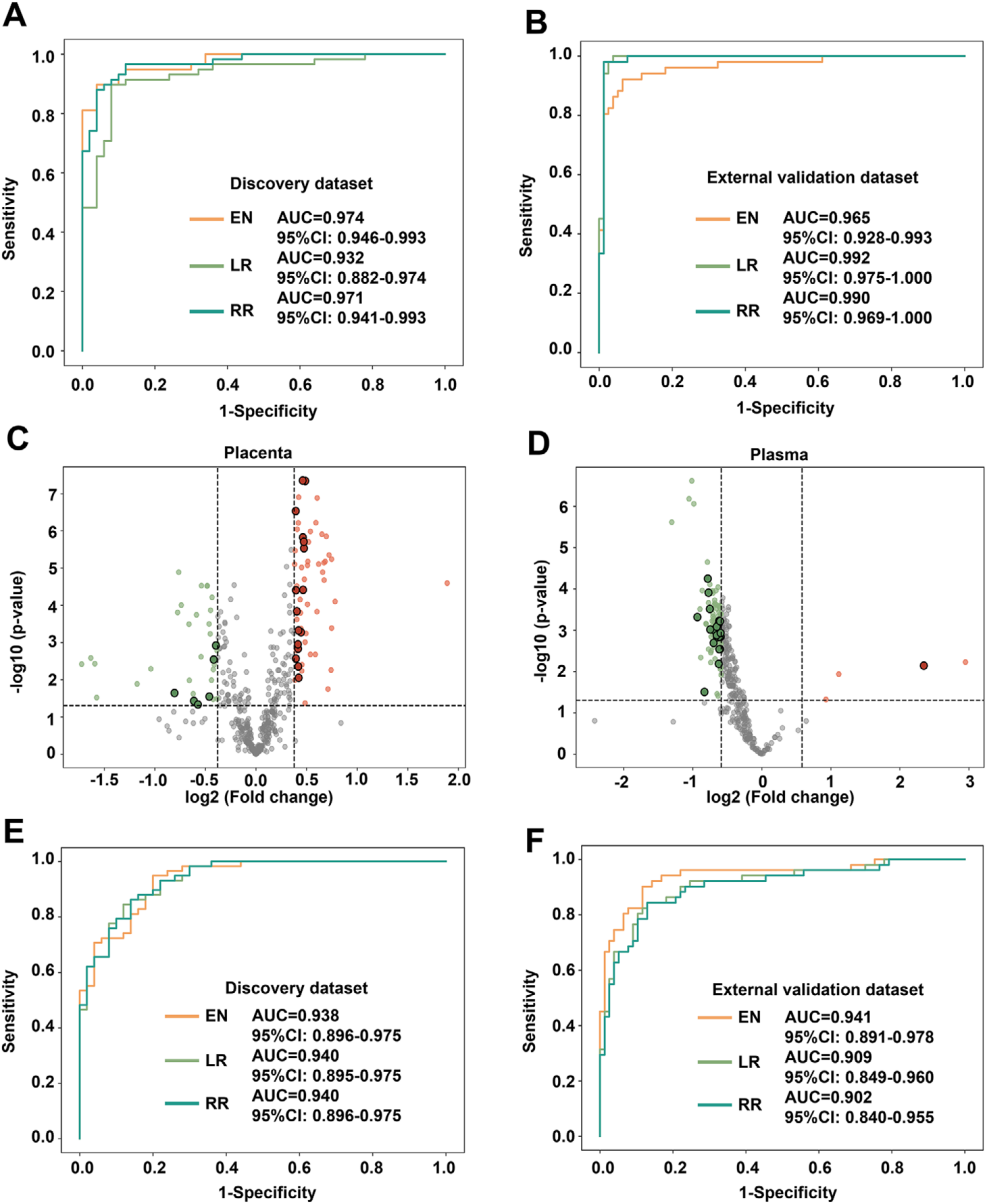
Construction of machine learning models for preeclampsia (PE) diagnosis based on plasma metabolic fingerprints. The receiver‐operating characteristic (ROC) curves generated by elastic net (EN), lasso regression (LR), and ridge regression (RR) based on plasma metabolic fingerprints with areas under the curve (AUC) of (A) 0.932‐0.974 in the discovery dataset and (B) 0.965–0.992 in the external validation dataset. Volcano plot for (C) placental and (D) plasma signals with fold‐change thresholds (|log_2_(FC)| of 0.58 in plasma and 0.38 in placental tissue, vertical dashed lines) and *p* value thresholds (*p* = 0.05 by Student's *t* test, horizontal dashed line), filtering 22 common signals between plasma and placenta metabolic fingerprints. The ROC curves generated by EN, LR, and RR based on 22 selected features, with AUC of (E) 0.938–0.940 in the discovery dataset and (F) 0.902–0.941 in the external validation dataset.

To support clinical translation and simplify diagnostic workflows, we performed feature selection based on the shared metabolic features from plasma and placenta provided by 37 PE patients and 33 non‐PE participants in the discovery dataset. Specifically, volcano plot analysis identified differentially expressed *m/z* signals between PE and non‐PE groups (Figure 3C,D). From the initial 351 plasma and 430 placental *m/z* signals, 22 common *m/z* features were retained based on fold‐change thresholds and statistical significance (*p* < 0.05 by Student's *t* test, Table S6). Models trained on these 22 features demonstrated robust diagnostic performance, with AUCs of 0.938–0.940 in the discovery dataset and 0.902–0.941 in the validation dataset (Figure 3E,F, Table S7). Sample‐level plot and PR curve analyses confirmed these results, with AUPRC consistently exceeding 0.880 across all models and datasets, highlighting an optimal balance between precision and recall (Figure S4). These results underscore the diagnostic value of the selected metabolic feature set.

### 2.4 Annotation of Metabolic Biomarker Panel

To further promote clinical applicability, we established a metabolic biomarker panel consisting of 19 metabolites putatively annotated from the 22 candidates and developed a final diagnostic model for PE. Specifically, the biomarker panel comprised 19 metabolites, including taurine (Tau), L‐alanine (Ala), succinimide (Sucm), 5‐amino‐2‐methoxyphenol (5A2MP), L‐valine (Val), pyridoxamine 5'‐phosphate (PMP), histidine (His), hypoxanthine (Hx), urolithin C (Uro‐C), orotic acid (OA), D‐alanyl‐D‐alanine (D‐Ala‐D‐Ala), L‐3‐hydroxykynurenine (3‐HK), d‐ribose 5‐phosphate (R5P), L‐cystathionine (Cysta), 2‐hydroxychlorpropamide (2HCPA), 1‐methylinosine (1‐MetIno), mannitol (Man), cyclic melatonin (cMel), and monoisobutyl phthalic acid (MiBPA, Figure 4A, Table S8).

**FIGURE 4:**
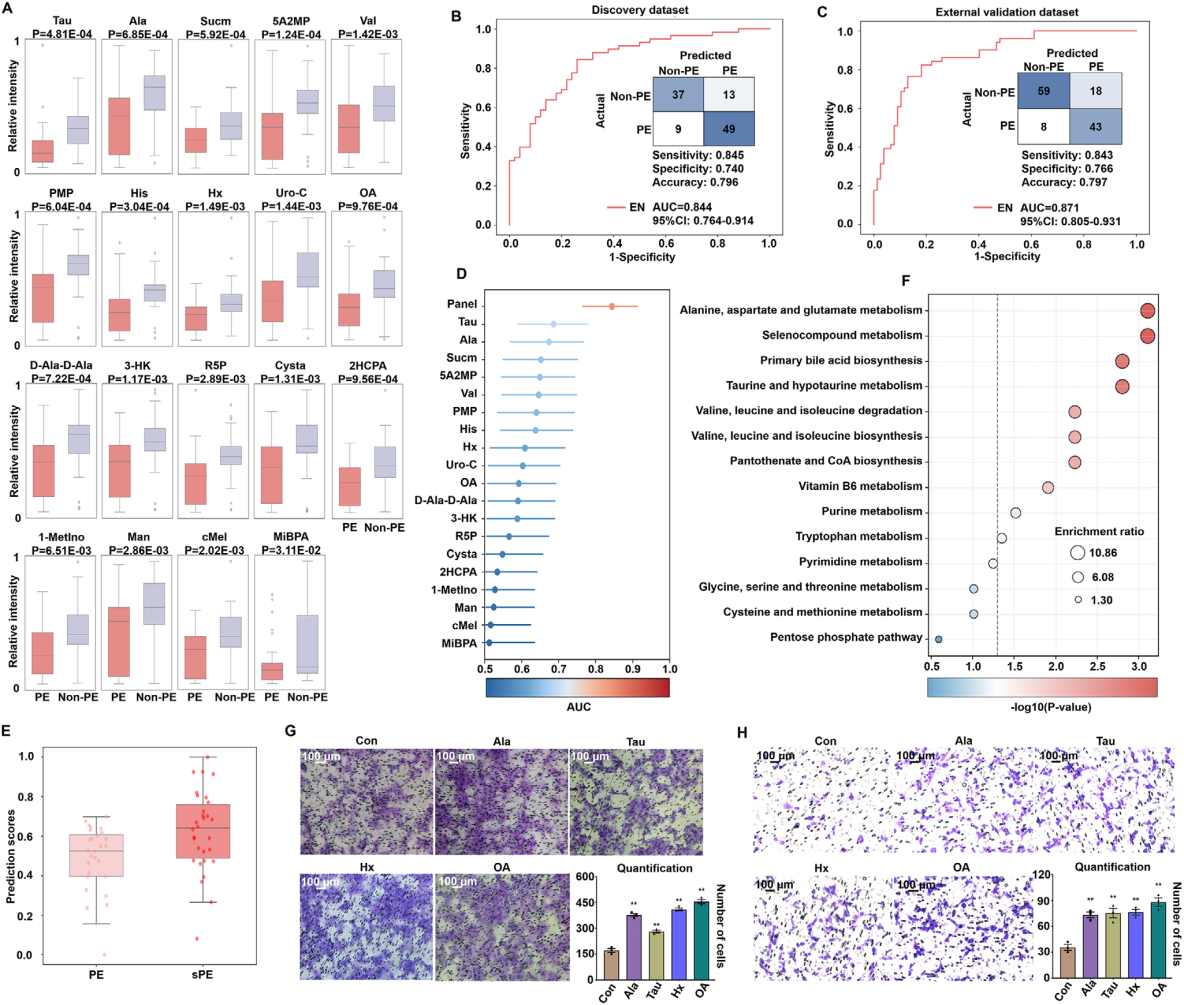
Annotation of metabolic biomarker panel and pathway analysis. (A) The differential expression of 19 metabolites, including taurine (Tau), L‐alanine (Ala), succinimide (Sucm), 5‐amino‐2‐methoxyphenol (5A2MP), L‐valine (Val), pyridoxamine 5'‐phosphate (PMP), histidine (His), hypoxanthine (Hx), urolithin C (Uro‐C), orotic acid (OA), D‐alanyl‐D‐alanine (D‐Ala‐D‐Ala), L‐3‐hydroxykynurenine (3‐HK), d‐ribose 5‐phosphate (R5P), L‐cystathionine (Cysta), 2‐hydroxychlorpropamide (2HCPA), 1‐methylinosine (1‐MetIno), mannitol (Man), cyclic melatonin (cMel), and monoisobutyl phthalic acid (MiBPA), in preeclampsia (PE) patients (pink) and non‐PE participants (purple), and *p* values were marked. ROC curves and confusion matrices of EN model based on the constructed metabolic panel in (B) discovery and (C) external validation dataset. (D) Enhanced AUC of the metabolic biomarker panel compared with individual biomarkers (*p* < 0.05, by DeLong test). (E) The box plot indicated different prediction scores given by the EN model of PE patients and sPE patients (*p* = 0.003, by Student's *t* test). (F) The plot of metabolite pathways revealed through quantitative enrichment analysis. The color and size of the circles indicated the *p* value by hypergeometric test and enrichment ratio, respectively. (G) Trophoblast cells HTR‐8/SVneo and (H) JAR were treated with different metabolites, including L‐alanine, taurine, hypoxanthine and orotic acid, with *p* values of *p* < 0.001, *p* = 0.003, *p* < 0.001, *p* < 0.001 for HTR and *p* < 0.001, *p* = 0.001, *p* < 0.001, *p* < 0.001 for JAR by Student's *t* test. The migratory capacity of the cells treated with different metabolites was significantly higher than that of the negative control, as determined by Transwell assays (values are mean ± SEM; *p < 0.05; **p < 0.01).

Furthermore, we evaluated the diagnostic efficacy of this biomarker panel in both datasets using the EN algorithm. In the discovery dataset, the model achieved a sensitivity up to 84.5% (95% confidence interval (CI): 73.1%–91.6%), specificity up to 74.0% (95% CI: 60.4%–84.1%), and AUC of 0.844 (95% CI: 0.764–0.914, Figure 4B). Similar results were obtained in the external validation dataset, with sensitivity reaching 84.3% (95% CI: 72.0%–91.8%), specificity up to 76.6% (95% CI: 66.0%–84.7%), and AUC of 0.871 (95% CI: 0.805–0.931, Figure 4C). Permutation test (1000 permutations) yielded *p* < 0.01 which indicated that our model captured meaningful relationships rather than random patterns (Figure S5A). Decision curve analysis (DCA) demonstrated that using the proposed model for decision‐making yielded greater net benefit than either treating all cases or treating none across a wide range of threshold probabilities (Figure S5B). For samples from Nantong First People's Hospital and Huai'an First People's Hospital, the model achieved AUC of 0.845 and 0.844, respectively, displaying generalizability among different clinical centers (Figure S5C). Multivariable logistic regression analysis adjusted for maternal clinical characteristics yielded *p* < 0.01, which proved the robustness of the associations between our biomarker panel and PE (Table S9). The biomarker panel demonstrated superior discriminatory power compared with established biomarkers (sFlt‐1, PlGF, and sFlt‐1/PlGF ratio) with *p* < 0.001 (Figure S5D) and with any individual metabolite with *p* < 0.05 by DeLong test (Figure 4D, Table S10). Moreover, patients with severe preeclampsia (sPE) exhibited significantly higher prediction scores (*p* = 0.003 by Student's *t* test) than those with PE (Figure 4E), demonstrating the model's capacity to differentiate disease severity.

Furthermore, we conducted quantitative enrichment analysis (QEA) to elucidate the biological relevance of the 19 metabolites in the panel. Ten metabolic pathways associated with PE were identified with *p* < 0.05 by hypergeometric test, including: (1) alanine, aspartate and glutamate metabolism; (2) selenocompound metabolism; (3) primary bile acid biosynthesis; (4) taurine and hypotaurine metabolism; (5) valine, leucine and isoleucine degradation; (6) valine, leucine and isoleucine biosynthesis; (7) pantothenate and CoA biosynthesis; (8) vitamin B6 metabolism; (9) purine metabolism; and (10) tryptophan metabolism (Figure 4F, Table S11).

Four metabolites that showed concordant downregulation in plasma and placental tissues—L‐alanine, taurine, hypoxanthine, and orotic acid—were selected for functional validation (Supporting Information). To investigate the effects of these metabolites on the migratory capacities of trophoblast cells, the migration abilities of HTR‐8/SVneo and JAR cells under treatment with various metabolites were analyzed. The results demonstrated that intervention at specified concentrations (1.0 mM L‐alanine, 10.0 mM taurine, 100 µM hypoxanthine, and 500 µM orotic acid) significantly enhanced the migration capabilities of both HTR‐8/SVneo and JAR cells (*p* < 0.01 by Student's *t* test) [31, 32, 33, 34] (Figure 4G,H), demonstrating that these metabolites can promote the migratory abilities of extravillous trophoblasts.

## 3 Discussion

PE is one of the leading causes of maternal and perinatal mortality, and its considerable clinical burden underscores the need to optimize diagnostic strategies [35]. Current PE diagnostic criteria, based on clinical signs such as hypertension and proteinuria, suffer from observer error and demonstrate poor ability to predict adverse outcomes [36]. False‐positive results compound these challenges, triggering unnecessary iatrogenic burdens, whereas false negatives overlook at‐risk patients requiring intensified surveillance [37]. Though emerging blood biomarkers (e.g., sFlt‐1/PlGF) offer improved accuracy, their focus on upstream pathways may miss subtle metabolic shifts inherent to PE's pathophysiology. Thus, developing diagnostic tools from the perspective of metabolomics is promising for high‐performance PE detection, even though routine obstetric implementation will require further prospective validation, inter‐laboratory standardization, automated data processing, and formal evaluation of cost‐effectiveness. Typically, increasing evidence now supports the notion that PE is not merely a vascular disorder but also a metabolic disease, driven by the essential metabolic reprogramming required for maternal adaptation throughout pregnancy [38, 39]. Thus, discovery of PE‐associated metabolic biomarkers is not only promising for accurate diagnosis, but also helps provide metabolic insight into PE progression. Previous metabolomic studies of PE have revealed metabolic adaptations and alterations associated with the condition, but these studies remain limited by dataset design and analytical platform [40, 41].

For analytical platforms, conventional metabolomic platforms (e.g., LC–MS/GC–MS) used in prior studies require extensive sample preparation and suffer from limited analytical throughput [40, 42, 43]. To address these limitations, we implemented NPELDI‐MS—a high‐performance platform that provides fast analytical speed of 2 s/sample by minimizing sample preparation through nanoparticle‐enabled size‐selective metabolic enrichment from complex biological samples [44, 45], while achieving high‐throughput (384 samples/batch) via microarray processing. This approach enabled high‐performance metabolic fingerprinting of 236 plasma and 70 placental tissue samples. The platform's robustness was further evidenced by high intra‐group cosine similarity over 0.75 across multicenter samples, addressing a key reproducibility issue in multicenter metabolomic studies.

Besides, robust dataset design is essential for reliable biomarker discovery and generalizable diagnostic models. While previous PE studies predominantly used single‐center datasets with single sample types (typically plasma or serum), our multicenter study enrolled participants across four clinical centers in two Chinese provinces and collected both plasma and placental tissue samples for biomarker discovery [40, 46]. A 19‐metabolite panel was derived from metabolic features consistently dysregulated in both sample types, which demonstrated robust diagnostic performance, achieving AUC of 0.844 (95% CI: 0.764–0.914) in the discovery dataset (two centers) and maintained consistent efficacy with AUC of 0.871 (95% CI: 0.805‐0.931) in an independent validation dataset (two additional centers), confirming population generalizability. Moreover, the biomarker panel exhibited significant diagnostic advantages compared with currently established biomarkers for PE (sFlt‐1, PlGF, sFlt‐1/PlGF ratio) with *p* < 0.001 by DeLong test.

Beyond diagnostics, our study also revealed significant perturbations across 10 key metabolic pathways in PE, some of which were consistent with previous reports and further highlighted the profound systemic dysregulation characteristic of PE. Taurine, found to be down‐regulated in plasma in our study, is a key component in antioxidant defense, osmoregulation, and membrane stabilization [47], which may contribute significantly to the oxidative stress, endothelial dysfunction, and altered lipid handling characteristic of PE. Specifically, its role in promoting the migratory abilities of extravillous trophoblasts was also validated in cell‐line experiments in our study. Besides, valine, as a branched‐chain amino acid, is strongly associated with insulin resistance and endothelial dysfunction, which are core features of PE pathophysiology according to a previous report [48]. The observed dysregulation of R5P in the pentose phosphate pathway is potentially linked to altered NADPH production (critical for redox balance and biosynthesis) and R5P supply for nucleotide synthesis, which affect the normal energy supply and redox balance in the body, contributing to the occurrence and development of PE [49]. These dysregulated metabolites and their corresponding pathway disturbances collectively reflect the systemic metabolic dysregulation in PE. Moreover, these putatively annotated metabolites captured the multi‐pathway dynamics, providing not only a better diagnostic tool but also important mechanistic insights for understanding the pathogenesis of PE.

We established a multicenter PE‐associated metabolic fingerprint database using NPELDI‐MS, comprising plasma and placental samples from 236 subjects. We putatively annotated a biomarker panel of 19 metabolites that achieved diagnostic AUCs of 0.844 (95% CI: 0.764–0.914) and 0.871 (95% CI: 0.805–0.931) in the discovery and independent validation dataset, respectively. Further quantitative enrichment analysis revealed dysregulated pathways related to PE, which provided mechanistic insights into PE pathogenesis. This work advances high‐throughput metabolic diagnostics for PE with significant potential for clinical translation. However, several limitations should be acknowledged. The biological mechanisms underlying PE‐associated metabolic alterations remain to be clarified. In addition, the predictive value of these biomarkers at earlier gestational stages should be assessed in prospective longitudinal studies. Further prospective validation, inter‐laboratory standardization, automated data processing, and cost‐effectiveness evaluation will also be needed before routine clinical implementation.

## 4 Materials and Methods

### 4.1 Study Dataset and Sample Collection

This retrospective study established a preeclampsia (PE) dataset comprising 236 subjects, including 109 PE patients (60 diagnosed as sPE) and 127 non‐PE participants. These participants were recruited from four clinical centers across two provinces in China, with enrollment occurring between January 2023 and December 2024.

For plasma preparation, venous blood was collected without prior fasting. In the discovery dataset, samples were collected during late pregnancy within 24 h before delivery, at a mean gestational age of 35.7 ± 2.7 weeks for PE patients and 38.6 ± 0.9 weeks for non‐PE participants. In the external validation dataset, samples were collected during the second trimester, at a mean gestational age of 21.7 ± 3.0 weeks for PE patients and 22.6 ± 2.7 weeks for non‐PE participants. The samples were then centrifuged at 1500 *g* for 5 min, and the resulting supernatant was collected for analysis. The resulting plasma supernatant was carefully aspirated, avoiding disturbance of the buffy coat layer, and then subjected to a second centrifugation at 10,000 *g* for 10 min at 4°C to remove residual platelets. Aliquots of the clarified plasma were rapidly frozen in cryovials and stored at −80°C to prevent degradation.

Placental tissue samples were collected promptly after delivery. Representative sections, approximately 1 cm^3^ in size, were excised from regions such as the placental attachment site, the periphery of the umbilical cord insertion point, and the placental parenchyma, while carefully avoiding areas with visible calcification, infarction, or necrosis. These samples underwent thorough rinsing in cold phosphate‐buffered saline (PBS) to remove residual blood. After rinsing, excess moisture was gently blotted away using sterile gauze. Samples were immediately snap‐frozen in liquid nitrogen and transferred to long‐term storage at −80°C. Frozen placental tissue samples were thawed at 4°C before 50 mg of each sample was weighed. The tissue was minced with scissors, ensuring no cross‐contamination between samples by wiping the scissors with alcohol and drying them with dust‐free paper after each use. The minced tissue was transferred into pre‐chilled microtubes, followed by the addition of 100 µL ultrapure water. Steel beads were added to each tube, and the samples were ground at 4°C for 2 min. The homogenate was then centrifuged at 12,000 *g* for 20 min at 4°C. The supernatant was carefully aspirated into a new centrifuge tube, with as much of the extracted solution as possible collected. A 50 µL aliquot of the supernatant was transferred to a new labeled centrifuge tube, and 50 µL of a 1:1 (v/v) methanol–acetonitrile mixture was added. The tube was vortexed for 1 min to mix thoroughly and then incubated at −20°C for 30 min. Finally, the mixture was centrifuged again at 12,000 *g* for 20 min at 4°C, and the resulting supernatant was transferred to a new centrifuge tube for subsequent mass spectrometry analysis.

According to current international guidelines (such as ACOG), a patient with preeclampsia—defined as new‐onset hypertension after 20 weeks of gestation—is diagnosed as severe PE with severe features if any of the following clinical or laboratory findings are present: severe‐range hypertension characterized by a systolic blood pressure of 160 mm Hg or greater, or a diastolic blood pressure of 110 mm Hg or greater on two occasions at least 4 h apart (or within a shorter interval if antihypertensive therapy is initiated); thrombocytopenia with a platelet count below 100,000/µL; impaired liver function indicated by serum transaminase levels elevated to twice the upper limit of normal, or severe, persistent right upper quadrant or epigastric pain; progressive renal insufficiency demonstrated by a serum creatinine concentration exceeding 1.1 mg/dL or a doubling of baseline levels; the development of pulmonary edema; or new‐onset, persistent cerebral or visual disturbances such as severe headaches, blurred vision, or scotomata.

### 4.2 Statistical Analysis

QEA was performed using MetaboAnalyst 5.0 (http://www.metaboanalyst.ca/) to elucidate the biological relevance of the putatively annotated metabolites during pathway analysis, with the Kyoto Encyclopedia of Genes and Genomes (KEGG) database utilized as the reference library for pathway mapping. The intra‐group similarity of metabolic fingerprints was evaluated by the cosine algorithm, and similarity scores were calculated using “scikit‐learn” package in Python. Differences between receiver operating characteristic (ROC) curves were assessed using the “DeLongUI‐master” function in MATLAB (version R2023b). The three unsupervised methods, t‐distributed stochastic neighbor embedding (t‐SNE), principal component analysis (PCA), and uniform manifold approximation and projection (UMAP), were implemented using “scikit‐learn” and “umap” packages in Python. For all statistical analyses, the significance level (*p* value) was set at 0.05.

## Author Contributions

*Conceptualization*: Yida Huang, Yifan Lian, Kun Qian, and Zhiwei Liu. *Data curation*: Yetao Xu, Yuchen Liu, Kun Xu, and Xiaokang Deng. *Formal analysis*: Yuchen Liu, Kejia Cao, Junjie Guo, Yanxi Yang, and Yanyan Li. *Funding acquisition*: Yetao Xu, Yida Huang, and Kun Qian. *Investigation*: Yuchen Liu, Kun Xu, and Xiaokang Deng. *Project administration*: Yida Huang, Yifan Lian, Kun Qian, and Zhiwei Liu. *Methodology*: Yuchen Liu, Jiao Wu, and Juxiang Zhang. *Resources*: Yetao Xu, Kun Xu, Xiaokang Deng, Huihui Ju, Xiaoyan Zhou, Weichun Tang, Ying Jiang, Yifan Lian, and Zhiwei Liu. *Supervision*: Yida Huang, Yifan Lian, Kun Qian, and Zhiwei Liu. *Writing – original draft*: Yetao Xu, Yuchen Liu, Kun Xu, and Xiaokang Deng. *Writing – review and editing*: Yetao Xu, Yuchen Liu, Yida Huang, and Kun Qian.

## Funding

The authors gratefully acknowledge financial support from Projects 92557304, 82502580, 8251001087, 82571966, 82421001, 82371696, 82301933, 82271742, and W2511090 by NSFC, Medical‐Engineering Joint Funds of Shanghai Jiao Tong University (YG2026QNB48, YG2025QNB57, and YG2024ZD07), Noncommunicable Chronic Diseases‐National Science and Technology Major Project (2026ZD0554401), the China Postdoctoral Science Foundation (BX20250372, 2025T180645, GZC20251513, and 2026M793205), and Shanghai Post‐doctoral Excellence Program (2024451 and 2025803). This work was also sponsored by Jiangsu Province Hospital High‐level Talent Cultivation Program (Phase I, CZ0121002010037), Jiangsu Province Maternal and Child High Quality Development Project (GZL2514), and Application Foundation Project of Changzhou Science and Technology Bureau (CJ20245039).

## Ethics Statement

This study was approved by the Medical Ethical Committee of Jiangsu Province Hospital (2021‐SR‐055), and was conducted in strict accordance with the Declaration of Helsinki. Informed consent was obtained from all participants in the study.

## Conflicts of Interest

Both the technology and the method of detecting bio‐samples were patented by the authors.

## Supporting information




**Supporting File**: mco271139‐sup‐0001‐SuppMat.docx

## Data Availability

The data supporting this study's findings are available from the corresponding author upon reasonable request.
