# Supplementary material for: Efficient Plasma and Placenta Metabolic Fingerprints for Preeclampsia Diagnosis: A Multicenter Study
Source: MedComm (2020). 2026 Nov 8;7(11):e71139. doi: 10.1002/mco2.71139 (PMC13744648; doi:10.1002/mco2.71139)
Supplement: Supplementary file 1 [file MCO2-7-e71139-s001.docx]

Supporting Information

**Efficient plasma and placenta metabolic fingerprints for preeclampsia diagnosis: a multicenter study**

**Author**

*Yetao Xu^1,2,3#^, Yuchen Liu^4,5#^, Kun Xu^6#^, Xiaokang Deng^7#^, Kejia Cao^4,5^, Junjie Guo^4,5^, Jiao Wu ^4,5^, Juxiang Zhang^4,5^, Yanxi Yang^4,5^, Yanyan Li^4,5^, Huihui Ju^8^, Xiaoyan Zhou^9^, Weichun Tang^10^, Ying Jiang^11^, Yida Huang^4,5^*, Yifan Lian^12^*, Kun Qian^4,5^*, and Zhiwei Liu^1,2^*.*

**Affiliations**

^1^Department of Obstetrics and Gynecology, International Peace Maternity and Child Health Hospital, School of Medicine, Shanghai Jiao Tong University, Shanghai, 200030, China.

^2^Shanghai Key Laboratory of Embryo Original Diseases, Shanghai, 200030, China.

^3^Department of Obstetrics and Gynecology, First Affiliated Hospital with Nanjing Medical University, Nanjing 210029, China.

^4^State Key Laboratory of Systems Medicine for Cancer, School of Biomedical Engineering, Institute of Medical Robotics and Shanghai Academy of Experimental Medicine, Shanghai Jiao Tong University, Shanghai 200030, China

^5^Division of Cardiology, Renji Hospital, School of Medicine, Shanghai Jiao Tong University, Shanghai 200127, China

^6^Department of Oncology, Jiangsu Cancer Hospital, The Affiliated Cancer Hospital of Nanjing Medical University, Jiangsu Institute of Cancer Research, Nanjing 210009, China.

^7^Wuxi Maternity and Child Health Care Hospital, Affiliated Women's Hospital of Jiangnan University, Wuxi 214002, China

^8^Changzhou Maternal and Child Health Care Hospital, Nanjing Medical University, Changzhou 213000, China.

^9^Department of Obstetrics, The Affiliated Huai'an No. 1 People's Hospital of Nanjing Medical University, Huaian 223300, China.

^10^Department of Obstetrics, Affiliated Hospital of Nantong University, Nantong First People's Hospital, Nantong 226000, China.

^11^Department of Obstetrics, Women's Hospital, Zhejiang University School of Medicine, Hangzhou 310006, China.

^12^Department of Gastroenterology, The Second Affiliated Hospital of Fujian Medical University, Quanzhou 362000, China.

*^#^* Yetao Xu, Yuchen Liu, Kun Xu, and Xiaokang Deng contributed equally to the work.

*^*^* Yida Huang, Yifan Lian, Kun Qian, and Zhiwei Liu shared co-corresponding authorship.

**Email:** [huangyida@sjtu.edu.cn](mailto:huangyida@sjtu.edu.cn), yifanlink@fjmu.edu.cn, [k.qian@sjtu.edu.cn](mailto:k.qian@sjtu.edu.cn), [ipmch-liuzhiwei@sjtu.edu.cn](mailto:ipmch-liuzhiwei@sjtu.edu.cn).

**Contents**

1. Supplementary Figures (1-5)

2. Supplementary Tables (1-11)

3. Methods

4. References

**1. Supplementary Figures**

**Figure S1 | (A)** Scanning electron microscopy image of ferric nanoparticles, showcasing their precisely controlled surface roughness. **(B)** Dynamic light scattering and **(C)** zeta potential measurements were performed in triplicate. **(D)** Optical extinction spectrum of the ferric nanoparticles, with the dashed purple line marking 355 nm—the wavelength of the Nd:YAG laser used in NPELDI-MS. **(E)** Digital image shows the microarray containing dried sample-nanoparticle mixtures with 5 mm spacing. **(F)** Signal intensities and coefficients of variation (CVs) for standard metabolites, including valine (Val), lysine (Lys), glucose (Glc), and sucrose (Suc).

**Figure S2 | Comparative evaluation of ferric NPs and alternative matrices for NPELDI-MS. (A)** UV–Vis absorption spectra of ferric NPs, CeO₂ NPs, Ce-MOF, and Au NPs. The shaded region indicates the wavelength around the 355-nm laser excitation used for NPELDI-MS. **(B)** Representative matrix-only mass spectra showing background signals generated by the four matrices in the low-*m/z* range. **(C, D)** Signal intensities (**C**) and signal-to-noise ratios (**D**) of four representative metabolites, including valine (Val), lysine (Lys), glucose (Glc), and sucrose (Suc), analyzed using the four matrices at 1 mg/mL (*n* = 5). **(E)** Representative NPELDI-MS spectra of a plasma sample acquired using the four matrices. **(F)** Total ion counts of plasma metabolic fingerprints obtained using the indicated matrices (*n* = 5). **(G)** Spot-to-spot and batch-to-batch coefficients of variation (CVs) for Val, Lys, Glc, and Suc measured using the four matrices (*n* = 5). **(H)** Representative spectra of Val, Lys, Glc, and Suc prepared in PBS at 1 mg/mL and analyzed using the four matrices to evaluate salt tolerance. Dashed lines indicate the characteristic *m/z* signals of the representative metabolites. **(I)** Overall comparison of the performance of the four NPELDI matrices across the evaluated analytical and practical characteristics.

**Figure S3 | Machine learning performance based on plasma metabolic fingerprints of preeclampsia (PE) patients and non-PE participants.** Sample level plot of **(A)** elastic net (EN), **(B)** lasso regression (LR), and **(C)** ridge regression (RR). Areas under precision-recall curves (AUPRC) in **(D)** discovery dataset and **(E)** external validation dataset.

**Figure S4 | Machine learning performance based on the shared 22 features of preeclampsia (PE) patients and non-PE participants.** Sample level plot of **(A)** elastic net (EN), **(B)** lasso regression (LR), and **(C)** ridge regression (RR). Areas under precision-recall curves (AUPRC) in **(D)** discovery dataset and **(E)** external validation dataset.

**Figure S5 | (A)** The model was retrained from scratch 1,000 times, and the AUC of the original model in the external validation dataset was significantly higher than that of all permuted models (*p* < 0.01). **(B)** Decision curve analysis demonstrated that our model achieved a higher net benefit than the "treat-all" and "treat-none" strategies across wide threshold probability range. **(C)** Diagnostic performance evaluation of the final biomarker panel for each center, with areas under the curve (AUC) of 0.845 (95%CI: 0.754-0.917) from Nantong First People's Hospital and 0.844 (95%CI: 0.744-0.928) from Huai'an First People's Hospital. **(D)** The diagnostic AUCs of the established biomarkers for PE (sFlt-1, PlGF, sFlt-1/PlGF ratio) and the machine learning model based on the constructed biomarker panel. The biomarker panel demonstrated superior discriminatory power compared with sFlt-1, PlGF, and sFlt-1/PlGF ratio with *p* < 0.001 by DeLong test.

**2. Supplementary Tables**

**Table S1 | The coefficients of variation (CVs) of signals of the standard metabolites independent of the sample test.**

| **MS signals ^a)^** | ***m/z*** | **Average intensity** | **Std** | **CV** |
| --- | --- | --- | --- | --- |
| [Val+Na]^+^ | 140.1 | 12560.1 | 1548.5 | 12.3% |
| [Lys+Na]^+^ | 169.1 | 15484.9 | 1576.7 | 10.2% |
| [Glc+Na]^+^ | 203.1 | 3667.2 | 446.7 | 12.2% |
| [Suc+Na]^+^ | 365.1 | 5487.9 | 585.4 | 10.7% |

^a)^ Val, valine; Lys, lysine; Glc, glucose; Suc, sucrose.

**Table S2 | The coefficients of variation (CVs) of signals of the standard metabolites during the sample test.**

| **MS signals ^a)^** | ***m/z*** | **Average intensity** | **Std** | **CV** |
| --- | --- | --- | --- | --- |
| [Val+Na]^+^ | 140.1 | 272901.5 | 53386.6 | 19.6% |
| [Lys+Na]^+^ | 169.1 | 517808.5 | 79082.0 | 15.3% |
| [Glc+Na]^+^ | 203.1 | 224746.4 | 38473.8 | 17.1% |
| [Suc+Na]^+^ | 365.1 | 808222.4 | 104823.6 | 13.0% |

^a)^ Val, valine; Lys, lysine; Glc, glucose; Suc, sucrose.

**Table S****3 | Clinical characteristics of the participants in the discovery dataset.**

| **Clinical indexes** | **Class** | **Discovery dataset** | | |
| --- | --- | --- | --- | --- |
|  |  | **PE patients**  **(n = 58)** | **non-PE participants**  **(n = 50)** | ***p* value** |
| Gestational age at delivery (weeks) | \ | 35.8±2.8 | 38.8±1.0 | <0.001 |
| Gestational age at sample collection (weeks) | \ | 35.7±2.7 | 38.6±0.9 | <0.001 |
| Age (years) | \ | 29.2±5.5 | 28.3±4.1 | 0.37 |
| BMI (kg/m²) | \ | 30.1±5.0 | 27.0±3.3 | <0.001 |
| Intrapartum hemorrhage (mL) | \ | 309.5±163.9 | 274.0±126.7 | 0.22 |
| Postpartum hemorrhage within 24 hours (mL) | \ | 437.3±184.8 | 379.4±130.0 | 0.07 |
| Apgar_1 min | \ | 9.5±1.3 | 9.9±0.4 | 0.03 |
| Apgar_5 min | \ | 9.8±0.6 | 10.0±0.0 | 0.09 |
| Birth weight (g) | \ | 2709.5±774.9 | 3322.2±324.3 | <0.001 |
| WBC (×10^9^/L) | \ | 10.9±3.1 | 9.5±2.6 | 0.02 |
| HGB (g/L) | \ | 114.3±13.6 | 110.3±13.4 | 0.12 |
| PLT(×10^9^/L) | \ | 182.2±65.8 | 201.9±47.1 | 0.08 |
| Parity (%) | 0 | 27(46.6) | 42(84.0) | <0.001 |
|  | 1 | 24(41.4) | 7(14.0) |  |
|  | 2 | 7(12.1) | 1(2.0) |  |
| Gravidity (%) | 1 | 27(46.6) | 36(72.0) | 0.05 |
|  | 2 | 15(25.9) | 5(10.0) |  |
|  | 3 | 9(15.5) | 7(14.0) |  |
|  | 4 | 5(8.6) | 1(2.0) |  |
|  | 5 | 1(1.7) | 0(0.0) |  |
|  | 6 | 1(1.7) | 1(2.0) |  |
| Assisted reproductive technology (%) | No | 58(100.0) | 50(100.0) | NA |
| Mode of delivery (%) | Vaginal delivery | 15(25.9) | 33(66.0) | <0.001 |
|  | Cesarean section | 43(74.1) | 17(34.0) |  |
| Neonatal transfer to pediatrics (%) | Yes | 25(43.1) | 1(2.0) | <0.001 |
|  | No | 33(56.9) | 49(98.0) |  |
| Medication use | Yes (Aspirin) | 39(67.2) | 6(12.0) |  |
|  | No | 19(32.8) | 44(88.0) |  |
| Diabetes | No | 58(100.0) | 50(100.0) |  |
| Preexisting hypertension | No | 58(100.0) | 50(100.0) |  |

**Table S4 | Clinical characteristics of the participants in the external validation dataset.**

| **Clinical indexes** | **Class** | **External validation dataset** | | |
| --- | --- | --- | --- | --- |
|  |  | **PE patients**  **(n = 51)** | **non-PE participants**  **(n = 77)** | ***p* value** |
| Gestational age at delivery (weeks) | \ | 35.8±2.6 | 38.7±1.2 | <0.001 |
| Gestational age at sample collection (weeks) | \ | 21.7±3.0 | 22.6±2.7 | 0.08 |
| Age (years) | \ | 29.5±3.4 | 33.5±3.4 | <0.001 |
| BMI (kg/m²) | \ | 32.0±5.1 | 27.2±4.1 | <0.001 |
| Intrapartum hemorrhage (mL) | \ | 286.9±121.4 | 224.9±78.2 | 0.001 |
| Postpartum hemorrhage within 24 hours (mL) | \ | 413.9±155.9 | 341.9±85.8 | 0.001 |
| Apgar_1 min | \ | 9.6±0.8 | 10.0±0.2 | <0.001 |
| Apgar_5 min | \ | 9.9±0.4 | 10.0±0.0 | 0.02 |
| Birth weight (g) | \ | 2859.6±723.2 | 3411.7±358.7 | <0.001 |
| WBC (×10^9^/L) | \ | 10.2±2.4 | 10.7±2.8 | 0.26 |
| HGB (g/L) | \ | 115.2±11.8 | 115.5±12.3 | 0.90 |
| PLT(×10^9^/L) | \ | 227.3±63.5 | 192.7±51.0 | 0.001 |
| Parity (%) | 0 | 36(70.6) | 16(20.8) | <0.001 |
|  | 1 | 10(19.6) | 40(51.9) |  |
|  | 2 | 4(7.8) | 17(22.1) |  |
|  | 3 | 1(2.0) | 4(5.2) |  |
| Gravidity (%) | 0 | 24(47.1) | 11(14.3) | <0.001 |
|  | 1 | 14(27.5) | 36(46.8) |  |
|  | 2 | 3(5.9) | 16(20.8) |  |
|  | 3 | 4(7.8) | 8(10.4) |  |
|  | 4 | 3(5.9) | 5(6.5) |  |
|  | 5 | 1(2.0) | 0(0.0) |  |
|  | 6 | 1(2.0) | 1(1.3) |  |
|  | 8 | 1(2.0) | 0(0.0) |  |
| Assisted reproductive technology (%) | Yes | 0(0.0) | 6(7.8) | 0.08 |
|  | No | 51(100.0) | 71(92.2) |  |
| Mode of delivery (%) | Vaginal delivery | 5(9.8) | 70(90.9) | <0.001 |
|  | Cesarean section | 46(90.2) | 7(9.1) |  |
| Neonatal transfer to pediatrics (%) | Yes | 10(19.6) | 1(3.6) | 0.09 |
|  | No | 41(80.4) | 27(96.4) |  |
| Medication use | Yes (Aspirin) | 31(60.8) | 4(5.2) |  |
|  | No | 20(39.2) | 73(94.8) |  |
| Diabetes | No | 51(100.0) | 77(100.0) |  |
| Preexisting hypertension | No | 51(100.0) | 77(100.0) |  |

| **Models** ^a)^ | **Discovery dataset** | | | | **External validation dataset** | | | |
| --- | --- | --- | --- | --- | --- | --- | --- | --- |
|  | **AUC**  **(95% CI)** | **Sensitivity**  **(95% CI)** | **Specificity**  **(95% CI)** | **Accuracy**  **(95% CI)** | **AUC**  **(95% CI)** | **Sensitivity**  **(95% CI)** | **Specificity**  **(95% CI)** | **Accuracy**  **(95% CI)** |
| EN | 0.974  (0.946-0.993) | 89.7%  (81.4%-96.6%) | 96.0%  (89.8%-100.0%) | 92.6%  (88.0%-97.2%) | 0.965  (0.928-0.993) | 86.3%  (76.3%-95.7%) | 94.8%  (89.5%-98.8%) | 91.4%  (85.9%-96.1%) |
| LR | 0.932  (0.882-0.974) | 89.7%  (81.2%-96.6%) | 92.0%  (84.1%-98.2%) | 90.7%  (85.2%-95.4%) | 0.992  (0.975-1.000) | 84.3%  (73.5%-93.5%) | 98.7%  (95.8%-100.0%) | 93.0%  (88.3%-96.9%) |
| RR | 0.971  (0.941-0.993) | 96.6%  (91.1%-100.0%) | 88.0%  (78.2%-96.0%) | 92.6%  (87.0%-97.2%) | 0.990  (0.969-1.000) | 94.1%  (86.7%-100.0%) | 98.7%  (95.8%-100.0%) | 96.9%  (93.8%-99.2%) |

**Table S5 | The diagnostic performance of machine learning models based on plasma metabolic fingerprints.**

^a)^ The machine learning models include elastic net (EN), lasso regression (LR), and ridge regression (RR).

**Table S6 | Fold-change (FC) and *p*-value of the 22 shared features.**

| **Index** | ***m/z*** | **Placenta** | | **Plasma** | |
| --- | --- | --- | --- | --- | --- |
|  |  | **FC** | ***p* value** | **FC** | ***p* value** |
| 1 | 134.1 | 0.75 | 0.003 | 0.65 | <0.001 |
| 2 | 144.0 | 0.73 | 0.03 | 0.65 | <0.001 |
| 3 | 194.1 | 0.76 | 0.001 | 0.66 | 0.001 |
| 4 | 200.1 | 1.33 | 0.001 | 0.58 | <0.001 |
| 5 | 202.0 | 0.65 | 0.04 | 0.52 | <0.001 |
| 6 | 205.1 | 1.36 | <0.001 | 5.08 | 0.007 |
| 7 | 212.9 | 0.67 | 0.05 | 0.64 | 0.001 |
| 8 | 216.1 | 1.34 | 0.004 | 0.59 | <0.001 |
| 9 | 232.0 | 1.34 | 0.009 | 0.59 | <0.001 |
| 10 | 232.9 | 0.57 | 0.02 | 0.61 | <0.001 |
| 11 | 237.0 | 1.40 | <0.001 | 0.65 | <0.001 |
| 12 | 258.9 | 1.38 | <0.001 | 0.66 | 0.003 |
| 13 | 266.9 | 1.38 | <0.001 | 0.56 | 0.03 |
| 14 | 274.9 | 1.34 | <0.001 | 0.62 | 0.002 |
| 15 | 288.8 | 1.32 | 0.003 | 0.65 | 0.001 |
| 16 | 298.9 | 1.39 | <0.001 | 0.64 | 0.001 |
| 17 | 300.9 | 1.39 | <0.001 | 0.66 | 0.001 |
| 18 | 304.9 | 1.33 | 0.001 | 0.65 | 0.007 |
| 19 | 306.9 | 1.31 | <0.001 | 0.65 | 0.003 |
| 20 | 314.8 | 1.31 | <0.001 | 0.60 | <0.001 |
| 21 | 316.8 | 1.32 | <0.001 | 0.64 | <0.001 |
| 22 | 324.8 | 1.38 | <0.001 | 0.66 | <0.001 |

**Table S7 | The diagnostic performance of machine learning models based on the 22 shared features selected by volcano-plot-based method.**

| **Models** ^a)^ | **Discovery dataset** | | | | **External validation dataset** | | | |
| --- | --- | --- | --- | --- | --- | --- | --- | --- |
|  | **AUC**  **(95% CI)** | **Sensitivity**  **(95% CI)** | **Specificity**  **(95% CI)** | **Accuracy**  **(95% CI)** | **AUC**  **(95% CI)** | **Sensitivity**  **(95% CI)** | **Specificity**  **(95% CI)** | **Accuracy**  **(95% CI)** |
| EN | 0.938  (0.896-0.975) | 94.8%  (88.5%-100.0%) | 80.0%  (67.9%-90.0%) | 88.0%  (82.4%-93.5%) | 0.941  (0.891-0.978) | 90.2%  (81.8%-97.8%) | 85.7%  (76.9%-93.3%) | 87.5%  (82.0%-93.0%) |
| LR | 0.940  (0.895-0.975) | 84.5%  (74.6%-93.2%) | 88.0%  (78.4%-96.0%) | 86.1%  (79.6%-92.6%) | 0.909  (0.849-0.960) | 82.4%  (70.9%-92.9%) | 88.3%  (80.9%-94.9%) | 85.9%  (79.7%-91.4%) |
| RR | 0.940  (0.896-0.975) | 86.2%  (76.4%-94.3%) | 86.0%  (75.6%-94.6%) | 86.1%  (79.6%-92.6%) | 0.902  (0.840-0.955) | 84.3%  (73.3%-94.2%) | 87.0%  (78.9%-93.9%) | 85.9%  (79.7%-91.4%) |

^a)^ The machine learning models include elastic net (EN), lasso regression (LR), and ridge regression (RR)

**Table S8 | The annotated 19 metabolic biomarkers.**

| **NPELDI-MS m/z** | **FTICR-MS accurate m/z** | **Formula** | **Adduct** | **Mass error (ppm)** | **Putatively annotated metabolite** | **LC-MS support** | **LC-MS/MS peaks** |
| --- | --- | --- | --- | --- | --- | --- | --- |
| 134.056 | 134.0192 | C3H7NO2 | [M+2Na−H]^+^ | 2.96 | L-Alanine | HILIC-NEG | 44.99617;44.99748;45.00077;72.00819;88.03690;88.03874;88.03966;88.04058;88.04150;88.04242 |
| 143.975 | 144.0035 | C4H5NO2 | [M+2Na−H]^+^ | 2.27 | Succinimide | NA | NA |
| 194.059 | 193.9988 | C5H11NO2 | [M+2K−H]^+^ | 4.24 | L-Valine | RP-POS | 43.05343;56.04888;58.02876;69.03378;72.07900;82.06400;98.05980;100.07585 |
| 201.987 | 201.9347 | C2H7NO3S | [M+2K−H]^+^ | 4.83 | Taurine | HILIC-POS | NA |
| 212.91 | 212.9585 | C5H4N4O | [M+2K−H]^+^ | 4.46 | Hypoxanthine | HILIC-NEG | 42.00025;65.99977;80.02520;108.01959;133.01755;151.02464 |
| 216.075 | 215.9831 | C7H9NO2 | [M+2K−H]^+^ | 3.25 | 5-Amino-2-methoxyphenol | NA | NA |
| 232.042 | 231.9897 | C6H9N3O2 | [M+2K−H]^+^ | 4.97 | Histidine | RP-POS | 42.03293;81.06721;95.08689;111.00574;138.06191 |
| 232.873 | 232.9364 | C5H4N2O4 | [M+2K−H]^+^ | 0.92 | Orotic acid | HILIC-NEG | NA |
| 236.996 | 237.0048 | C6H12N2O3 | [M+2K−H]^+^ | 4.08 | D-Alanyl-D-alanine | NA | NA |
| 258.888 | 258.9993 | C6H14O6 | [M+2K−H]^+^ | 4.76 | Mannitol | HILIC-NEG | 55.02061;59.01535;71.01272;73.02992;78.95809;101.02369;163.06275 |
| 266.932 | 267.0604 | C12H14O4 | [M+2Na−H]^+^ | −0.05 | Monoisobutyl phthalic acid | RP-POS | 65.03690;93.03216;121.02819;149.02271 |
| 274.854 | 275.0775 | C13H14N2O2 | [M+2Na−H]^+^ | 2.86 | Cyclic melatonin | RP-NEG | NA |
| 288.822 | 289.0081 | C13H8O5 | [M+2Na−H]^+^ | −0.89 | Urolithin C | RP-NEG | NA |
| 298.86 | 298.9854 | C7H14N2O4S | [M+2K−H]^+^ | −3.47 | L-Cystathionine | HILIC-NEG | 36.96636;41.00499;44.01461;59.01075;73.04070;89.02087;91.05403;94.97989;103.05631;106.03891;130.98230;147.04324;148.99303;164.07042;221.09281 |
| 300.856 | 300.9973 | C10H12N2O4 | [M+2K−H]^+^ | −4.77 | L-3-Hydroxykynurenine | HILIC-NEG | 57.97787;70.98226;96.95825;138.96700;149.00783;178.98993;179.14339;205.06388;223.12987 |
| 304.867 | 305.0865 | C11H14N4O5 | [M+Na]^+^ | 2.82 | 1-Methylinosine | NA | NA |
| 306.878 | 306.9393 | C5H11O8P | [M+2K−H]^+^ | 3.57 | D-Ribose 5-phosphate | HILIC-NEG | NA |
| 314.813 | 315.019 | C10H13ClN2O4S | [M+Na]^+^ | 4.27 | 2-Hydroxychlorpropamide | NA | NA |
| 324.847 | 324.9749 | C8H13N2O5P | [M+2K−H]^+^ | −0.98 | Pyridoxamine 5′-phosphate | RP-NEG | NA |

**Table S9 | Association between the 19-metabolite panel and preeclampsia after adjustment for maternal clinical characteristics.**

| **Variable** | **Adjusted odds ratio per SD** | **95% CI** | ***p* value ^a)^** |
| --- | --- | --- | --- |
| 19-metabolite panel | 5.375 | 2.850-10.137 | <0.001 |
| 19-metabolite panel + GA | 6.086 | 2.572-14.404 | <0.001 |
| 19-metabolite panel + Age | 5.260 | 2.797-9.891 | <0.001 |
| 19-metabolite panel + Parity | 5.677 | 2.857-11.281 | <0.001 |
| 19-metabolite panel + Gravidity | 5.236 | 2.779-9.868 | <0.001 |
| 19-metabolite panel + BMI | 5.065 | 2.625-9.773 | <0.001 |
| 19-metabolite panel + Medication | 4.595 | 2.326-9.076 | <0.001 |
| 19-metabolite panel + GA + Age + Parity + Gravidity + BMI + Medication | 6.923 | 1.980-24.211 | 0.002 |

^a)^ *p*-values are generated by Wald test.

**Table S10 | The diagnostic AUCs of identified 19 metabolic biomarkers and the elastic net machine learning model based on the constructed metabolic panel.**

| **Metabolite** | **AUC** | **95% CI** | ***p* value ^a)^** |
| --- | --- | --- | --- |
| L-alanine | 0.674 | 0.573-0.774 | <0.001 |
| Succinimide | 0.652 | 0.550-0.755 | <0.001 |
| L-valine | 0.646 | 0.543-0.749 | <0.001 |
| Taurine | 0.687 | 0.587-0.786 | <0.001 |
| Hypoxanthine | 0.609 | 0.503-0.715 | <0.001 |
| 5-amino-2-methoxyphenol | 0.649 | 0.546-0.752 | <0.001 |
| Histidine | 0.638 | 0.534-0.741 | <0.001 |
| Orotic acid | 0.592 | 0.485-0.699 | <0.001 |
| D-alanyl-D-alanine | 0.590 | 0.483-0.697 | <0.001 |
| Mannitol | 0.525 | 0.415-0.634 | <0.001 |
| Monoisobutyl phthalic acid | 0.513 | 0.403-0.622 | <0.001 |
| Cyclic melatonin | 0.517 | 0.407-0.626 | <0.001 |
| Urolithin C | 0.603 | 0.497-0.709 | <0.001 |
| L-cystathionine | 0.548 | 0.439-0.657 | <0.001 |
| L-3-hydroxykynurenine | 0.589 | 0.482-0.696 | <0.001 |
| 1-methylinosine | 0.528 | 0.419-0.637 | <0.001 |
| D-ribose 5-phosphate | 0.566 | 0.458-0.674 | <0.001 |
| 2-hydroxychlorpropamide | 0.534 | 0.425-0.643 | <0.001 |
| Pyridoxamine 5'-phosphate | 0.640 | 0.536-0.744 | <0.001 |
| Panel | 0.844 | 0.764-0.914 | / |

^a)^ *p*-values are generated by DeLong test.

**Table S11 | The related 10 metabolic pathways revealed by quantitative enrichment analysis (QEA).**

| **Pathway** | ***p* value ^a)^** | **Enrichment ratio ^b)^** |
| --- | --- | --- |
| Alanine, aspartate and glutamate metabolism | <0.001 | 10.86 |
| Selenocompound metabolism | <0.001 | 10.86 |
| Primary bile acid biosynthesis | 0.002 | 9.66 |
| Taurine and hypotaurine metabolism | 0.002 | 9.66 |
| Valine, leucine and isoleucine degradation | 0.006 | 7.43 |
| Valine, leucine and isoleucine biosynthesis | 0.006 | 7.43 |
| Pantothenate and CoA biosynthesis | 0.006 | 7.43 |
| Vitamin B6 metabolism | 0.01 | 6.16 |
| Purine metabolism | 0.03 | 4.60 |
| Tryptophan metabolism | 0.04 | 4.01 |

^a)^ *p*-values are generated by hypergeometric test.

^b)^ The enrichment ratio was calculated as the ratio of observed to expected metabolite hits.

**3. Methods**

**3.1 Chemicals and reagents**

Ferric particles were prepared using ferric chloride hexahydrate (97%), trisodium citrate dihydrate (99%), anhydrous sodium acetate (99%), and ethylene glycol (99.5%), all of which were supplied by Sinopharm Chemical Reagent Beijing Co., Ltd. (Beijing, China). Regarding standard metabolites, L-alanine (Ala, 98%) and biological-grade L-lysine (Lys) were supplied by Sinopharm Chemical Reagent Beijing Co., Ltd. (Beijing, China). D-glucose (Glc, 99.7%) and sucrose (Suc, 99.7%) were obtained from Nanjing Chemical Reagent Co., Ltd. (Nanjing, China). Taurine (Tau, 99.88%) was purchased from MedChemExpress (New Jersey, USA), while hypoxanthine (Hx, 99.0%) and orotic acid (OA, 99.0%) were obtained from Sigma-Aldrich Trading Co.,Ltd (Shanghai, China). All chemicals and reagents were used as received without further purification. All aqueous solutions employed in the experiments were prepared with deionized water (18.2 MΩ·cm) produced by a Milli-Q system (Millipore, GmbH).

**3.2 Laser desorption/ionization mass spectrometry (LDI-MS) detection**

Metabolic fingerprinting of PE-associated plasma and placental tissue samples was performed using nanoparticle-enhanced laser desorption/ionization mass spectrometry (NPELDI-MS) with ferric nanoparticles as the matrix. These nanoparticles were synthesized through an optimized solvothermal approach. In this procedure, sequential dissolution of ferric chloride hexahydrate, trisodium citrate dihydrate, and anhydrous sodium acetate was performed in ethylene glycol solvent. The homogeneous mixture underwent 45 minutes of ultrasonic treatment at ambient temperature before being sealed in a Teflon-lined autoclave for solvothermal reaction at 200 °C for 10 hours. Post-synthesis purification involved extensive washing cycles using both ethanol and deionized water, culminating in a 24-hour drying process at 60 °C to eliminate all traces of solvent.

The NPELDI-MS experiments were carried out on an Autoflex mass spectrometer (Bruker Daltonics, Germany), equipped with an Nd:YAG laser operating at 355 nm wavelength. For detection, 1 μL of the analyte was carefully printed onto a microarray chip. The droplets were allowed to air dry. Subsequently, 1 μL of the ferric nanoparticle matrix slurry (prepared in deionized water at a concentration of 1.0 mg/mL) was applied onto the dried analyte spots. This streamlined sample preparation process enabled rapid sample analysis, achieving a speed of 2 seconds per sample (using 2,000 laser shots at 1,000 Hz) and 1 second for inter-sample switching. Experiments were conducted in positive ion mode. The obtained mass spectra were subjected to data preprocessing using a custom-built Python code (version 3.9), involving crucial steps such as filtering, baseline correction, peak detection, and peak alignment. Specifically, we applied Gaussian filtering for smoothing and morphological baseline correction to mitigate noise and artifacts. Peaks with a signal-to-noise ratio (S/N) ≥ 3 were detected using the SciPy package (version 1.10.1), and alignment was performed across all spectra, retaining only *m/z* features present in ≥2/3 of samples within each group to ensure reliability and reduce variability. After that, PE-associated plasma and placenta metabolic fingerprints were generated for further analysis. To ensure accurate MS measurement for biomarker annotation, Fourier transform ion cyclotron resonance mass spectrometry (FTICR-MS) was employed on a Solarix 7.0T instrument (Bruker Daltonics, Germany). Molecular formula confirmation was performed using Bruker Compass Data Analysis software (version 5.0) with an *m/z* mass tolerance of 10 ppm. Candidate metabolites were annotated by searching the Human Metabolome Database (HMDB). We substantially strengthened the metabolite annotation procedure by performing LC-MS/MS analysis as an orthogonal validation.

**3.3 Diagnostic model construction**

The construction of diagnostic models for PE from the metabolic fingerprints involved two primary steps: feature selection and model building. All machine learning analyses were conducted using Python with 'scipy' and 'scikit-learn' packages. To identify clinically relevant metabolic features, volcano plot analysis was employed to pinpoint differentially expressed *m/z* signals between the PE and non-PE groups. From an initial pool of 351 plasma and 430 placental *m/z* signals, 22 common *m/z* features were retained. This selection was based on stringent fold-change thresholds (|log2(FC)| > 0.58 in plasma, |log2(FC)| > 0.38 in placental tissue) and statistical significance (*p* < 0.05 by Student’s t test).

Three supervised machine learning algorithms were evaluated, including elastic net (EN), lasso regression (LR), and ridge regression (RR). For EN, the regularization parameter was set to α = 200 with an L1 ratio of 0.5, and the maximum number of iterations was 100. LR and RR were implemented using the SAGA solver, with the inverse regularization strength C = 0.01. The maximum number of iterations was 100 for LR and 300 for RR. The models were trained using the original feature values. Hyperparameters not explicitly described above were left at their library default values.

Models were trained on the discovery dataset and tested on the independent external validation dataset to assess their generalizability and robustness. The diagnostic performance of the plasma metabolic fingerprints was evaluated using receiver operating characteristic (ROC) curve analysis, yielding areas under the curve (AUC). Precision-recall (PR) curve was also utilized, with areas under the PR curve (AUPRC) serving as performance metrics.

**3.4 Cell culture**

HTR-8/SVneo cells were generously provided by Prof. Charles Graham (Queen's University, Kingston, ON, Canada). JAR was from the Institute of the Chinese Academy of Sciences (Shanghai, China). The culture conditions for HTR-8/SVneo and JAR cells were as follows: DMEM culture medium containing 10% FBS and 1% antibiotics (100 μg/mL streptomycin and 100 U/mL penicillin G), cultured in a humidified incubator at 37 °C with 5% CO2 and 90% humidity. The complete culture medium was replaced every 2–3 d depending on the cell growth status, and the cells were passaged and used for subsequent experiments when they reached 80–90% confluence.

**3.5 Transwell assays**

Cell migration assays were performed as previously reported^1^. Cells were treated with each metabolite, and Transwell assays were conducted after 24 h of treatment. 3-5×104 cells were cultivated on the top of a membrane for cell migration assays. Cells inside the upper chamber were removed after incubation for 24 h, 36 h, 48 h, and 60 h. Then cells on the lower membrane surface were fixed with methanol and stained with 0.5% Crystal violet solution. 3-5 randomly selected fields were counted per well.

**4. References**

1.	Deng X, Zuo Q, Liu Y, et al. Inflammation-induced TET3/mir-3942 axis impedes the proliferation and invasion ability of trophoblast cells through destabilization of SERPINE1 in preeclampsia. *Placenta*. 2025;167:193–203.
